# The effectiveness of mother-led infant massage on symptoms of maternal postnatal depression: A systematic review

**DOI:** 10.1371/journal.pone.0294156

**Published:** 2023-12-13

**Authors:** Orla Geary, Annmarie Grealish, Ann-Marie Bright

**Affiliations:** Department of Nursing and Midwifery, University of Limerick, Limerick, Ireland; University of Gothenburg: Goteborgs Universitet, SWEDEN

## Abstract

**Introduction:**

Postnatal depression is a significant public health issue which may escalate and lead to adverse outcomes for women, infants, their family and the wider society. The aim of this review was to examine the effectiveness and experiences of mother-led infant massage on symptoms of maternal postnatal depression and to synthesise these findings to inform policy, practice and further research.

**Methods:**

A systematic search of five academic databases was conducted: CINAHL, MEDLINE, EMBASE, PsycINFO and Allied and Complementary Medicine Database in February 2023 with no date or geographic limiters set owing to the paucity of research on this subject area. Quality appraisal was undertaken using the Joanna Briggs Institute quality appraisal tools and all included RCT’s were assessed separately using the Cochrane Risk of Bias Tool. Narrative synthesis was used to analyse the data.

**Findings:**

A total of (n = 323) studies were returned of which (n = 8) met the inclusion criteria for the review. This review identified a total sampling of (n = 521) women with maternal postnatal depression. The results are presented under three themes: 1) the effectiveness of mother-led infant massage on symptoms of postnatal depression; 2) women’s experiences of mother-led infant massage; and 3) the effects of mother-led infant massage on the mother-infant relationship.

**Discussion:**

The review highlights women who used infant massage displayed a reduction in symptoms of postnatal depression, improved mother-infant interactions and improved self-efficacy in addition to benefits for infants. Public Health Nurse/Community Midwife-led infant massage may help to relieve such symptoms and empower women.

## Introduction

The transition to motherhood is an extraordinary time that can place increased demands on women [1]. The perinatal period is defined as beginning at conception evolving until one year after birth [2,3]. During this time, women experience a merging of new roles and responsibilities with their pre-existing life, together with the burden of perceived social expectations on becoming a mother [4]. Most women will experience the “baby blues” within the first two weeks after birth characterised as an adjustment reaction [5]. This change in mood is transient but women are at increased risk of developing Postnatal Depression (PND) if the appropriate supports are not in place [5]. It is estimated that 11% to 29% of new mothers will experience symptoms of PND in Ireland [3]. These prevalence rates are echoed globally; in Australia it is estimated 1 in 7 women experience PND [6] with as many as 1 in 3 women continuing to report symptoms of depression for up to four years after birth [7]. In the United Kingdom (UK) it is estimated that 11% of women experience symptoms of depression in the first year post-birth [8], similar to estimations of 11% reported in the United States of America (USA) [9].

Well-known risk factors for developing PND include previous mental health issues [10], suboptimal social, partner and emotional support [11], low income [12] and exposure to previous trauma including traumatic birth [13]. PND consequently is a predictor for suicide [14] which is the principal cause of direct death for women within the perinatal period [15,16]. PND can also have detrimental effects on the wider family; children of women who experience PND are more likely to display disruptive behaviours, mental health issues [17], have poor health outcomes and developmental delay [18]. Partners may also experience the negative impact of PND often characterised by their own mental health issues, parental stress and relationship breakdown [19]. PND can have negative implications for society as a whole, with exorbitant costs associated with both child and maternal health [20].

Although healthcare services worldwide recognise perinatal mental health as a public health issue, barriers relating to the implementation of practice guidelines still exist [21–26]. Healthcare professionals should strive to promote positive wellbeing for women by enforcing a salutogenic approach in their everyday role [27]. Public Health Nurses (PHN’s), a position similar to Health Visitors in the UK, have a pivotal role in supporting women soon after discharge from the maternity services. Although PHNs may engage with women in the antenatal period, their primary encounter is in the postnatal period. PHNs and Health Visitors are in an ideal position to recognise any potential issues such as symptoms or predictors of postnatal depression [24,28]. However, screening and onward referral to additional agencies such as the General Practitioner, parent-infant services and social work is the primary remit of PHNs in relation to perinatal mental health [25].

There is an abundance of evidence pertaining to the use of non-pharmacological and complementary interventions for the treatment of PND [29–32]. Interventions in the postnatal period have demonstrated a positive effect on mothers mental wellbeing such as Cognitive Behavioural Therapy (CBT) [30,32] where a reduction in Edinburgh Postnatal Depression Scale (EPDS) [33] scores were reported in mothers who received CBT. Furthermore, a psychoeducation intervention formulated by Holt et al. [32] displayed a significant reduction in parental stress at six months. In their systematic review, Dixon and Dantas [34] examined a variety of interventions for PND that exist in a community setting. All studies included interventions provided by healthcare professionals who were not mental health trained and their results indicate that community interventions encompassing CBT, infant stimulation, or problem solving strategies prompted an overall improvement in mother’s wellbeing [34].

Complementary and alternative therapies are increasingly prevalent in preventing and in treating many conditions including mental health issues [35]. Lindensmith [36] examined previous studies and interventions focusing on the improvement of mother-infant interactions for women with PND. Interventions included the use of infant massage and the results indicated an improvement in mother-infant interactions, bonding and maternal attitude towards the infant which is congruent with previous research [37]. Further to this, ample research exists supporting the use of infant massage to improve infant outcomes such as jaundice levels [38], behavioural responses [39] and bonding, attachment and sleep [40]. In the context of PND, evidence supporting a reduction in postnatal depression for mothers is weak [36,37]. Lotfalipour et al. [31] observed a significant improvement in the mood of women massaging their pre-term infants however these results may not be applicable to women who gave birth to term infants due to the emotional impact premature birth can have on a woman [41].

Infant massage is a useful technique which can be used to help strengthen mother-infant interactions, facilitate additional social support for women [42] and improve infant health outcomes [43]. This is reflected in a Health Visitor-led infant massage course in the UK [44] and demonstrates the potential complementary role for PHNs in Ireland and internationally. Therefore, the introduction of infant massage classes facilitated by PHN’s may assist in the prevention, reduction or identification of symptoms of PND. Subsequently, the aims of this review were to examine the effectiveness and experiences of mother-led infant massage on symptoms of maternal postnatal depression and to synthesise these findings to inform policy, practice and further research.

## Methods

This systematic review was conducted using Cochrane guidance [45] and reported in accordance with the PRISMA statement [46] and is registered on the international prospective register of systematic reviews (PROSPERO: CRD42022307302). A Population, Intervention, Outcome (PIO) framework was adopted to create the selection of keywords in the search strategy and to formulate the following review question: “*What are the effects and experiences of mother-led infant massage on symptoms of maternal postnatal depression*?*”*

### Search strategy

An initial search of the Cochrane library, Open Grey database, Google Scholar and Prospero was completed to avoid duplication of research. This search yielded no current or recently published systematic reviews on the effectiveness of infant massage on PND. Subsequently, the following five electronic databases were searched in February 2023: Cumulative Index to Nursing and Allied Health Literature (CINAHL), MEDLINE, EMBASE, PsycINFO and Allied and Complementary Medicine Database (AMED). Owing to an overall lack of studies in this area and to ensure all relevant studies were incorporated, no date or geographical limiters were set. Due to time constraints, studies were limited to those published in the English language. A set of search terms was created using Medical Subject Headings (MeSH), thesaurus and associated free-text terms and were then combined using Boolean operators as shown in Table 1. Forward citation tracking was also completed through hand searching reference lists of selected studies.

**Table 1:** PIO framework.

PIO	Description	Search Terms
Population	Mothers experiencing symptoms of depression in postnatal period	**1.** mother* OR matern* OR parent* 2. “postnatal” OR post-natal OR postpartum OR puerperium OR "perinatal” OR after pregnancy OR after delivery OR after birth OR following birth **AND** depression OR depressive disorder OR depressive symptoms OR major depressive disorder OR “postpartum depression” OR postnatal depression OR PPD OR PND OR mood disorders OR mental health
Intervention	Infant Massage	infant massage OR “massage” OR newborn massage” OR “massage in infancy” OR massage education OR baby massage OR massage therapy OR Touch OR positive touch OR massage methods
Outcome	Reduction in symptoms of postnatal depression	effectiveness OR outcome OR symptom reduction OR “treatment” OR health outcome*

### Eligibility criteria

Studies were included if they reported peer-reviewed primary qualitative, quantitative or mixed method data related to the effects of infant massage on symptoms of maternal postnatal depression. Any study that detailed valid outcome measures for postnatal depression and perinatal mood disorders were included. Studies that sampled postnatal women over 18 years and in the postpartum period with term infants were included. Studies that did not report findings that related to the effects of infant massage on symptoms of maternal postnatal depression were excluded as were non-peer-reviewed articles, unpublished literature, consensus statements, discussion papers, opinion papers and expert reviews.

### Study selection

All studies retrieved in the database searches were combined and exported to the online platform Colandr [47], duplicates were removed and the remaining citations underwent title, abstract and full-text screening. Two authors (OG and AMB) assessed titles and abstracts against eligibility criteria and articles were excluded by agreement. Any disagreements were resolved through discussion with third author (AMG).

### Quality appraisal

The quality appraisal of each study was undertaken using the Joanna Briggs Institute (JBI) Checklist for Randomised Control Trials (RCTs) [48], the JBI Checklist for Quasi-Experimental Studies [47] and the JBI Checklist for Qualitative Research [49]. Each tool contains a series of questions which are rated “Yes”, “No”, “Unclear” or “N/A”. Risk of bias was conducted using the Cochrane Risk of Bias Tool [50] for all RCTs included in this review. Each RCT was appraised to either have high, low or uncertain risk of bias. The quality appraisal was considered in the presentation and discussion of results. Quality appraisals were conducted by two reviewers independently (OG and AMB and disagreements resolved through discussion with the advice of third reviewer (AMG).

### Data extraction

A Microsoft Word data extraction template was developed and used to tabulate methodological information from each study to include: author, year, country of origin, aim of study, infant massage intervention (delivery of intervention, women’s experiences of teaching sessions), sample characteristics, research setting, research design, outcome measures and key findings. The principal outcome of interest was a reduction in symptoms of maternal postnatal depression. Subordinate outcomes recorded were any additional benefits arising from infant massage i.e. quality of life, satisfaction levels, sleep quality, stress and mother-infant interactions. Any disputes during the study selection and data extraction processes were resolved through consultation between all three authors (OG, AMG & AMB).

### Data analysis and synthesis

A narrative synthesis informed by Popay et al. [51] was completed. This involved viewing textual descriptions extracted from relevant papers in tabulated form. This allowed the dataset to be visualised in its entirety. The review team then explored the dataset for patterns and similarities from within each individual study and across studies. These patterns and similarities were grouped, named and presented as themes. To reduce subjectivity, two authors (OG and AMB) contributed to the construction of each theme. Meta-analysis was not completed owing to the small number of included studies and the absence of statistical homogeneity.

## Results

### Search outcomes

**Fig 1:**
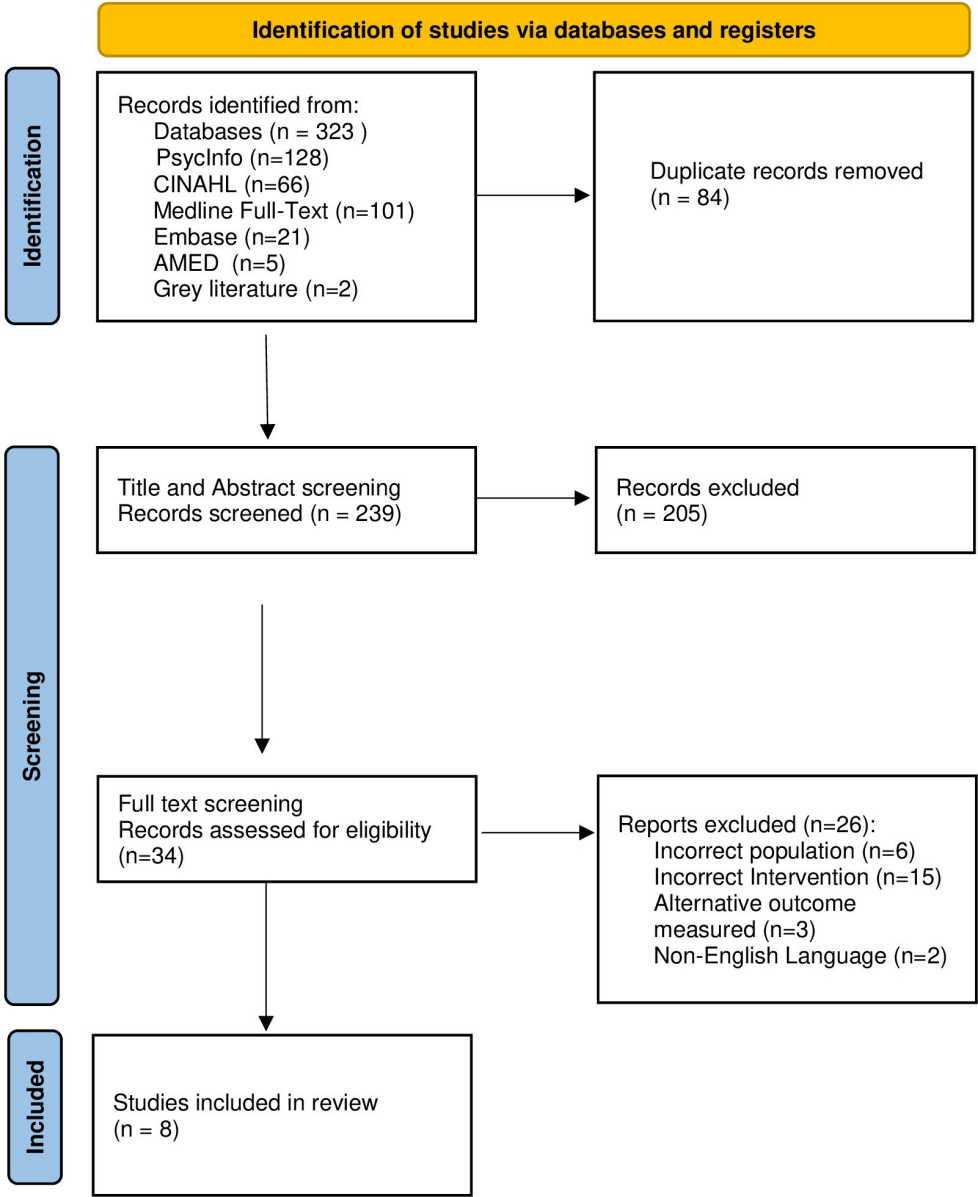
PRISMA flow diagram, Page et al. 2021 [46].

The literature search returned 323 studies. After the removal of duplicates (n = 84) and title and abstract screening, 34 studies were included for full-text screening from which eight (n = 8) met the eligibility criteria for this review. Fig 1 provides an overview of the study selection, screening and eligibility process.

### Study characteristics

Table 2 outlines the characteristics of the eight included studies. The returned studies were conducted between the period spanning March 2001–February 2019. The included studies sampled 521 participants collectively and individual sample sizes ranged from 9 to 180. One study was conducted in Japan [52], one in Malaysia [53], one in Iran [54], one in Norway [55], two in the UK [56,57] and two in the USA [58,59]. Study designs included three RCTs [54,56,78], two pilot RCTs [52,58], two qualitative exploratory studies [53,54] and one quasi-experimental study [57]. Studies recruited participants from hospital settings [21,56], local health clinics [53–55], HIV treatment centres [58] and research organisations [59]. One study did not specify the recruitment methods used [57]. All studies reported on the effects of infant massage on mothers [52–59].

All studies obtained ethical approval [52–59].

**Table 2:** Study characteristics.

Author, Year &Country	Study title	Aim & methodology	Data collection				Intervention type	Key Findings
Fujita et al. (2006) [52] Japan	Effect of massaging babies on mothers: pilot study on the changes in mood states and salivary cortisol level.	To evaluate the effects of baby massage for 3 months after delivery on mothers’ mood status and salivary cortisol level Quantitative–Experimental Pilot study RCT	Convenience sample N = 57 Mothers who delivered babies at the Tohoku Kosai Hospital in Japan from July to December 2001 Mean age of mother = 31.8 Mean age infant = 5.5 weeks Infant gender = not recorded Primiparous = (n = 9) Multiparous = (n = 10) Eliminated (n = 18) Experimental(n = 19) Control (n = 20) Psychological assessment: Questionnaire assessing demographics, Profile of Mood States (POMS) Data analysed using SPSS. The homogeneity of the two groups was analysed by the w2 -test and Student’s t-test. Comparisons between the experimental group and the control group at each of the points were analysed by the Wilcoxon rank sum test and Student’s t-test. The level of statistical significance was set at a P-value of .05. Statistical difference in both groups was non-significant The means and standard deviations (SD) for the baby massage time reported by the experimental group was 1072.9 min/day. The POMS was compared between the I group and C groups. No significant difference at baseline. 3 months after delivery scores improved more in depression and vigor in the experimental group compared to the control group (D; t = -2.57, P = 0.2, V; t = 2.39, P = 0.2). The tension, anger, fatigue, and confusion scores in the experimental group were slightly lower than those of the control group, but the difference did not reach statistical significance (T–A; t = -1:35, P = 19, A–H; t = 1.62, P = .12, F; t = -1.84, P = .08, C; t = -1.32, P = .20)				Infant massage Completed at home with instructions at beginning of study Intervention group encouraged to use infant massage as per Field’s method at least 10 minutes per day until 3 months postnatal, note each session, complete and return questionnaire before and after intervention and submit salivary cortisol samples prior to first day of infant massage and 3 months post-delivery. The control group was asked to reply to a questionnaire and to take salivary cortisol samples twice (5–6 weeks after delivery, and 3 months after delivery). All mothers were asked to mail the samples and questionnaires to the author within 24 h. The papaer does not state who taught/demonstrated the infant massage for mothers at the beginning of the study	Decrease in depression score after 3 months for intervention group. Additional benefits noted: Increase in vigor of infants of mothers in intervention group at 3 months.
Mindell et al. (2018) [59] USA	Massage-based bedtime routine: impact on sleep and mood in infants and mothers	To examine the impact of a massage-based bedtime routine on infant sleep, maternal sleep, and maternal mood. Quantitative RCT	N = 123 Intervention (n = 64) Control (n = 59) Mean maternal age = 30.6 Mean infant age = 8.96 months Mean infant gender = Male = 46.3 Female = 53.7 Parity = not recorded Demographic Questionnaire, Brief infant sleep questionnaire, Pittsburgh sleep quality index, Epworth sleepiness scale, EPDS, Brief mood introspection scale, Parenting stress index-short form, State-trait anxiety inventory (Form Y Descriptive analyses to describe variables Preliminary analyses (samples t-tests and chi-square tests) used to outline any demographic or sleep differences between control group and intervention group Univariate/ ANOVAS Pair wise comparisons Bonferroni correction McNemar analyses for dichotomized variables, comparing binomial distributions. IBM SPSS Statistics Version 22 software				Nightly two-step bedtime massage routine for a two weeks. Mothers were instructed to have lights out within 30 minutes after completing the full-body massage. This paper does not specify who provided instructions regarding infant massage strokes 59 families participated as controls. They continued regular bedtime routine. They were informed the study was looking at sleep problems. All questionnaires completed online.	Majority of mothers reported “somewhat” or “very satisfied” with massage based routine at the end of week one. No changes noted for EPDS scores, daytime sleepiness (ESS), parenting stress (PSI), state anxiety (STAI) Significant improvement in maternal mood (BMIS) from baseline to week 1 and week 2
Onozawa et al. (2001) [56] United Kingdom	Infant massage improves mother-infant interaction for mothers with postnatal depression	To determine whether attending regular massage classes could reduce maternal depression and also improve the quality of mother-infant interaction. Quantitative RCT	N = 34 Intervention (n = 12) Control (n = 13) Dropout Intervention (n = 7) Control (n = 2) Mean maternal age = 32 Mean infant age = 9 weeks Male infant = (n = 17) Female infant = (n = 8) Parity not recorded EPDS, Intervention group: Baseline—15, First session– 9.5 Final session—5.0 Control Group: Baseline—16.0, First session—13.0, Final session—10.0 Reduction in EPDS score from recruitment to the final session for the massage group was significantly greater than for the control group. Face to face play interaction–video recorded, Video recording assessed using observation measures–Global ratings for infant/mother interactions (Fiori-Cowley and Murray) Random assessment of 10 dyads carried out by independent assessor blind to study Dimensions examined: Maternal: (a) warm to cold, (b) non- intrusive to intrusive; Infant: (a) attentive to non- attentive, (b) lively to inert, (c) happy to distressed;. Interaction: smooth/difficult; fun/serious; satisfying/unsatisfying; much engagement/no engagement; excited engagement/quiet engagement Each dimension assessed displayed a significant improvement for the intervention group. With no change for the control group. Non- parametric method due to small sample size Comparisons carried out using Mann–Whitney U-test or Fisher’s exact probability				One hour long sessions of group infant massage classes for five weeks instructor demonstrated technique on doll. Instructor was IAIM trained however it was not specified who provided the classes to mothers. Mothers encouraged to focus on infant cues throughout class.	Significant reduction in EPDS scores in intervention group when compared with control group. Mother-infant interaction measured through video-taping improved significantly by end of study when compared with control.
O’ Higgins et al.(2008) [57] United Kingdom	Postnatal depression and mother and infant outcomes after infant massage	To investigate the benefits of infant massage for mothers with postnatal depression and their infants. Quantitative Quasi-experimental design	N = 96 Intervention (infant massage) (n = 31) Support group (n = 31) Non-depressed (n = 34) Mean age mother = (n = 18) Mean age infant = 4 weeks Infants gender not recorded Parity not recorded EPDS, Spielberger State Anxiety Inventory (SSAI), Infant Characteristics Questionnaire (ICQ), Global Ratings for Mother-Infant Interactions SSAI and EPDS for both depressed groups remained higher than those in the non-depressed group. Massage group EPDS scores were non-significantly lower than the support group.				6 sessions of instructor led infant massage group classes Sessions 1 hour long and were run by trained members of the International Association of Infant Massage, however it is not specified who offered these classes to mothers. Each class began with a group discussion and then focussed on different massage strokes demonstrated by the instructors on dolls. The emphasis was on paying attention to infant cues and responding appropriately so different massage strokes and amounts of massage would happen for each mother–infant pair and in each class. The support group was set up specifically for the research project and was run by an experienced research team member. All mothers in the study attended the 5 weekly support group sessions. The massage group and control group attended separately. The sessions involved informal discussions around practical advice and coping strategies.	SSAI and EPDS scores for both depressed groups remained higher than those in the non-depressed group. Massage group EPDS scores were non-significantly lower than the support group. 87% of massage group experienced a clinically significant reduction in EPDS scores when compared with support group (63%) The median score for the massage group was below the cut-off for possible depression at one year, unlike the support group.
			Mean scores	Massage group	Support Group	Non-depressed group		
			Baseline	EPDS 13.19 SSAI 44.7	EPDS 13.81 SSAI 45.49	EPDS 3.24 SSAI 12.01		
			6 weeks	EPDS 9.29 SSAI 37.6	EPDS 11 SSAI 37.97	EPDS 3.82 SSAI24.61		
			One year	EPDS 9.15 SSAI 36.08	EPDS 9.82 SSAI 39.58	EPDS 3.39 SSAI 25.54		
			87% of massage group experienced a clinically significant reduction in EPDS scores when compared with support group (63%) The median score for the massage group was below the cut-off for possible depression at one year, unlike support group. Mothers who completed 4 sessions or more as well as outcome measures were included ANOVAs with post hoc Bonferroni tests–compare groups. The interactions were rated using the Global Ratings for Mother–Infant Interactions by a blinded trained rater					
Midtsund et al. (2019) [55] Norway	Mothers experiences learning and performing infant massage–a qualitative study	To explore the experience of learning infant massage among mothers who are having insecurity and stress in their transition to motherhood A qualitative, explorative approach	Convenience sample N = 12 Mean maternal age = 34 Mean infant age = 4 months Infant gender not recorded Primiparous = (n = 7) Multiparous = (n = 5) Interviews–open-ended questions Interviews guided by open-ended questions Follow up questions were asked to increase the depth and clarify information received Qualitative content analysis method (Graneheim and Lundmans 2004) Meaning units gathered Coding, sub-categories and categories created Themes formulated Each of the above steps completed independently by each researcher initially then as a group to ensure high quality data extraction				Public Health Nurse led Infant massage classes, once a week for six weeks. massage aims to be a tool for improving interaction between mother and child. The infant massage is organized and arranged according to IAIM principles (McClure, 2010).	Infant massage helped mothers to feel calm and at ease Infant massage provided protected time with infant Mothers felt infant massage was for them as much as it was for infant. Infant massage made mothers feel more confident in there role. Infant massage helped resolve feelings of guilt Infant massage allowed mothers to feel more secure and positive
Oswalt and Biasini (2011) [58] USA	Effects of infant massage on HIV-infected mothers and their infants	To determine the feasibility of implementing an infant massage intervention and to evaluate the preliminary effects if infant massage on HIV-infected mothers and their infants Quantitative RCT	N = 17 Control (n = 9) Intervention (n = 8) Mean age mothers = 25.5 Mean age infant = 7.5 weeks Infant gender not recorded Parity not recorded Demographic questionnaire, Beck Depression Inventory-II, Maternal confidence Questionnaire, Parenting Stress Index-short form, Questionnaire about physical contact, Infant growth measurements All continuous demographic variables were analyzed using independent samples t-tests. Due to small sample size, study used power analysis software) to determine whether adequate power was available to detect significant differences Sufficient power for planned statistical tests was determined. Analysis of covariance was conducted to determine the significance of differences between the treatment and control groups, with pretreatment scores used as covariates in the analysis. Intention-to-treat analysis was conducted so that subjects were analysed based on the group that they were originally assigned				Mothers in the intervention group received 15–20 minute training session at the clinic after baseline assessments were taken. Mothers in the intervention group were asked to massage their infants once each day for 10 weeks The PI (Postdoctoral Research Associate) of this study was a certified instructor in the Baby’s First Massage program. The Baby’s First Massage program was taught to participants in the intervention group. The control group received routine care	Mothers in control group reported higher levels of depression than mothers in the intervention group post intervention. N = 3 mothers in control group report significant depression at follow up compared to N = 0 in intervention group **Depression Pre-test** 7.67 (Control) 8.38 (Intervention) **Depression Post-test** 8.61 (Control) 5.31 (Intervention)
Chan et al., 2018 [53] Malaysia	Experience of mothers’ learning and doing infant massage	To explore mothers’ experience with learning and doing infant massage.	N = 9 Mean maternal age = 26.6 Mean infant age = 5 weeks Infant gender not recorded Parity: Primiparous = (n = 6) Multiparous = (n = 3) Face to face semi structured interviews conducted in the participants chosen language Open-ended questions Audio recording of interviews transcribed verbatim Field notes taken to record additional information Data collection ceased once data saturation reached Thematic analysis guided by Creswell six generic steps for data analysis Coding Interpretation of participants experiences Inferential statistics not reported in this study				Weekly instructor led infant massage for four weeks. Mothers instructed to continue massage at home twice daily for 15 minutes An adapted infant massage program (McClure, 2015) was utilised and guided by a certified infant massage instructor (CIMI) in the clinic. No further details re instructor were provided.	Increased maternal satisfaction and confidence. Mothers were happy to learn new skills in relation to caring for their babies. Mothers appreciated peer support at the group and enjoyed a space to share experiences and receive advice from other mothers. Mothers reported strengthened bonding with their infant. Mothers reported infants were more relaxed and calm. Mothers learned how to interpret infant’s cues. Mothers reported increased partner support.
Dehkordi et al., 2018 [54] Iran	The Effects of infant Massage on Maternal Postpartum Depression: A Randomised Control Trial	To investigate the effects of infant massage by mothers on maternal PPD.	N = 120 Lost to dropout = (n = 60) Mothers age ranged between 18–45 Infants = 2 weeks approx. Male infant = (n = 33) Female infant = (n = 27) Parity not recorded Intervention and control group randomly allocated. PPD assessed using EPDS pre and post study. Mothers included if deemed to have PPD determined by a EPDS score of 10 or over. SPSS software Kolmogorov-Smirnov Chi-square test t-test Wilcoxon signed rank test Univariate logistic regression analysis Multivariable logistic regression Hosmer-Lemeshow test The level of significant for all tests set to <0.05				Mothers received a 15 minute demonstration of infant massage from the primary investigator if the study who is a registered infant massage instructor on day one of study, Each mother was then assessed performing infant massage. There on, mothers were instructed to continue infant massage at home twice per day for 6 consecutive weeks. Instructional materials provided. Weekly face to face or telephone support provided by researcher. Control group did not receive training in infant massage. No further details provided.	The mean EPDS score for intervention group was significantly lower after intervention. Mean scores pre-test Intervention 11.51 Control 11.31 Mean scores post-test Intervention 7.75 Control 9.20 *P* value <0.001

### Participant characteristics

The range of demographic data gathered differed greatly in each study. Maternal level of education was determined in three studies with majority of participants having received higher level/university education [53–55], while paternal level of education was determined in one study [54]. Marital status was recorded in four studies and ranged from single, married and living with partner [52–56]. Maternal parity was recorded in three studies [52,53,55] and sampled both primiparous and multiparous women. Infant’s gender was recorded in three studies [54,56,59] however, as not all studies reported gender in the same way, it was not possible to provide an aggregate. Four studies examined participant’s ethnicity with a wide range of ethnic backgrounds included, for example; White, Asian, Black, Hispanic and Multi-ethnic [53,56,58,59]. Further demographic details included mode of delivery, English speaking [56], infant birth measurements [58], employment status [53,54], transport [53], household income [53,54] and type of residence [54].

### Outcome measures

Similar outcome measures were utilised throughout the studies with some exceptions. To assess affective symptoms the EPDS [33], Brief Mood Introspection Scale (BMIS) [60], Profile of Mood States (POMS) [61] and the Beck Depression Inventory II (BDI II) [62] were utilised. To assess maternal confidence, the Maternal Confidence Questionnaire [63] was utilised and to assess anxiety the State Trait Anxiety Inventory (STAI) [64] was used. The Pittsburgh Sleep Quality Index (PSQI) [65] and the Epworth Sleepiness Scale (ESS) [66] were used to assess sleep quality. To assess mother-infant interactions the Global Ratings for Mother-Infant Interactions [67] was used. Outcome measures relevant to the infant included the Infant Characteristics Questionnaire (ICQ) [68] and Infant Growth Measurements.

### Characteristics of infant massage interventions

Studies included in this systematic review utilized various frameworks to implement infant massage. One study [58] used Ramsay’s [69] “baby’s first massage curriculum”, two studies [52,54] used Fields’ [70] “touch” mechanisms and three studies [55–57] used McClure’s [71] techniques from the International Association of Infant Massage. Chan et al. [53] and Mindell et al. [59] did not specify a chosen framework. The infant massage interventions varied in duration from two weeks to three months. Some were conducted in a structured class setting [53,55–57] while others were conducted in the home setting [52,54,58,59]. Six studies had a control group [52–54,56–59] and one did not [55]. One study offered an open-ended, practical support group to the control group which mothers could join at any stage [57]. Another study provided a support group to all participants including the control group, however both groups attended separately [56].

### Quality of studies

**Fig 2:**
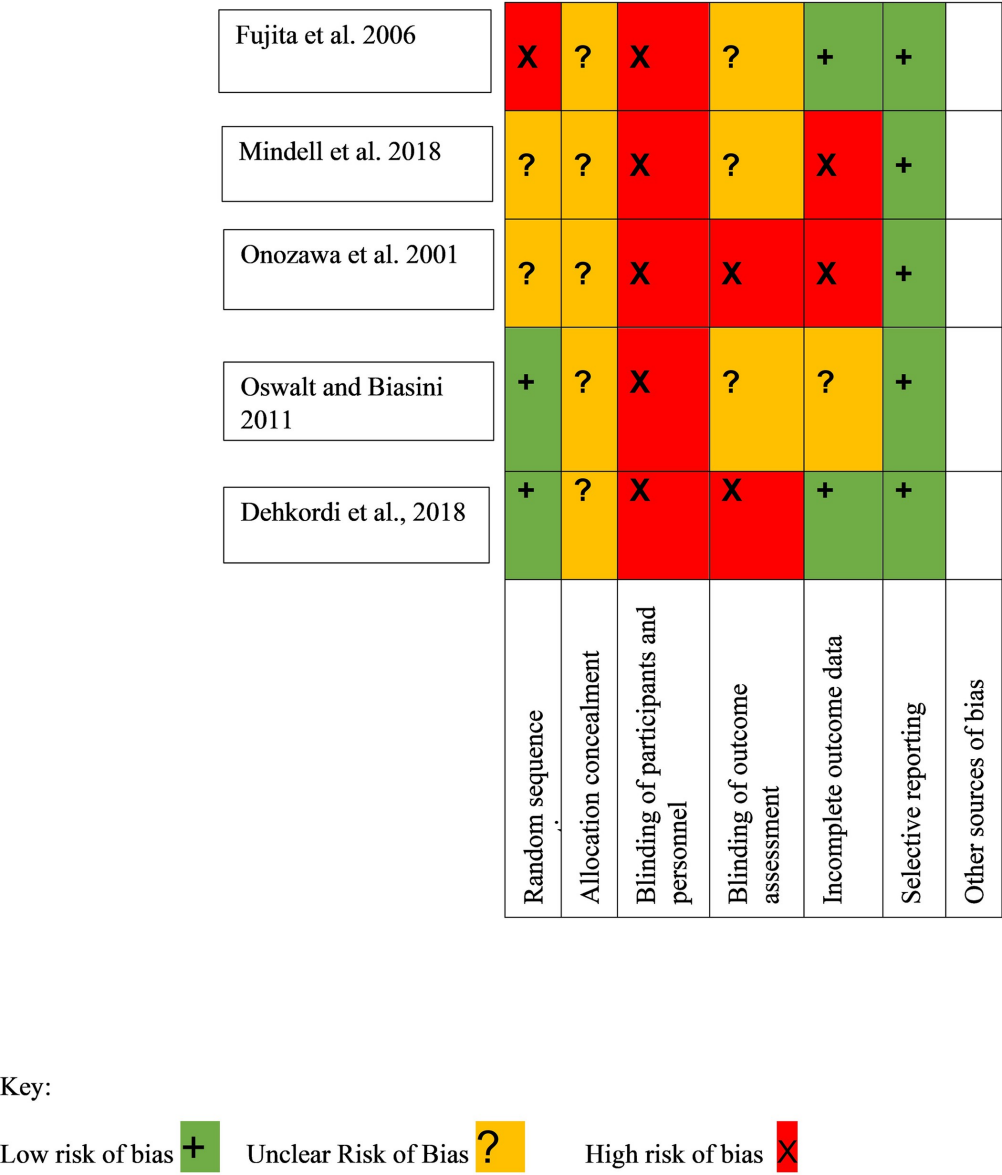
Risk of Bias (Higgins et al. 2011) [45].

Table 3 outlines the quality of the studies determined by the JBI Checklist for RCTs [48], the JBI Checklist for Qualitative Studies [49] and the JBI Checklist for Quasi-Experimental Studies [48]. Further quality appraisal was completed using GRADE [72]. Overall, the eight included studies were considered to be of fair quality and of high risk of bias (see Fig 2). Whilst the quality of all studies were mixed, studies were not excluded based on the quality assessment. Some studies lacked important methodological criteria and were deemed deficient in scientific rigour. For example, the presence of concealed allocation groups and participant and outcome assessor blinding is unclear in four of the six RCT’s [52,54,56,59].

**Table 3:** Quality appraisal.

Joanna Briggs Institute: Randomised Controlled Trial Checklist	Onozawa et al. (2001) [56]	Oswalt & Biasini (2011) [58]	Fujita et al. (2006) [52]	Mindell et al. (2018) [59]	Dehkordi et al. (2018) [54]
Was true randomisation used for assignment of participants to treatment groups?	Yes	Yes	Yes	Yes	Yes
Was allocation to treatment groups concealed?	Unclear	Unclear	Unclear	Unclear	Unclear
Were treatment groups similar at the baseline?	Yes	Yes	Yes	Unclear	Yes
Were participants blind to treatment assignment?	No	No	Unclear	Unclear	Unclear
Were those delivering treatment blind to treatment assignment?	No	Unclear	Unclear	Unclear	No
Were outcome assessors blind to treatment assignment?	Unclear	Unclear	Unclear	Unclear	Unclear
Were treatment groups treated identically other than the intervention of interest?	Yes	Yes	Yes	Yes	No
Was follow up complete and if not, were differences between groups in terms of their follow up adequately described and analyzed?	Yes	yes	Yes	Unclear	unclear
Were participants analyzed in the groups to which they were randomized?	Yes	Yes	Yes	Yes	Yes
Were outcomes measured in the same way for treatment groups?	Yes	Yes	Yes	Yes	Yes
Were outcomes measured in a reliable way?	Yes	Yes	Yes	Yes	Yes
Was appropriate statistical analysis used?	Yes	Yes	Yes	Yes	Yes
Was the trial design appropriate, and any deviations from the standard RCT design (individual randomization, parallel groups) accounted for in the conduct and analysis of the trial?	Unclear	Yes	Yes	Yes	Yes
**Joanna Briggs Institute: Critical Appraisal Checklist for Qualitative Research**	Midstund et al. (2019) [55]	Chan et al. (2018) [53]
Is there congruity between the stated philosophical perspective and the research methodology?	Unclear	Yes
Is there congruity between the research methodology and the research question or objectives?	Yes	Yes
Is there congruity between the research methodology and the methods used to collect data?	Yes	Yes
Is there congruity between the research methodology and the representation and analysis of data?	Yes	Yes
Is there congruity between the research methodology and the interpretation of results?	Yes	Yes
Is there a statement locating the researcher culturally or theoretically?	Yes	Yes
Is the influence of the researcher on the research, and vice- versa, addressed?	Yes	Yes
Are participants, and their voices, adequately represented?	Yes	Yes
Is the research ethical according to current criteria or, for recent studies, and is there evidence of ethical approval by an appropriate body?	Yes	Yes
Do the conclusions drawn in the research report flow from the analysis, or interpretation, of the data?	Yes	Yes
**Joanna Briggs Institute: Quasi- Experiemental Studies Checklist**	O’Higgins et al. (2008) [57]
Is it clear what is the ‘cause’ and what is the ‘effect’?	Yes
Were the participants included in any comparisons similar?	Yes
Were participants treated similarly other than the exposure or intervention?	Unclear
Was there a control group?	Yes
Were there multiple pre and post measurements of the outcome?	Yes
Was follow-up complete or were group differences adequately addressed?	Unclear
Were the outcomes included in any comparisons measured in the same way?	Unclear
Were outcomes measured in a reliable way?	Yes
Was appropriate statistical analysis used?	Yes
**Key**: Green = Yes, Red = No, Orange = Unclear	

### Review findings

The findings of this review will be discussed under the following themes: (1) the effectiveness of mother-led infant massage on reducing symptoms of postnatal depression; (2) women’s experiences of mother-led infant massage and; (3) the effects of mother-led infant massage on the mother-infant relationship.

### The effectiveness of mother-led infant massage on reducing symptoms of postnatal depression

All eight studies reported a reduction in symptoms of postnatal depression in women participating in infant massage. Fujita et al. [52] reported particularly on maternal mood post-infant massage. Baseline scores for each mood state as per the POMS for both intervention (I) and control (C) groups were as follows; tension-anxiety: I = 49.6, C = 50.9 (*p* = .66); depression-dejection: I = 46.3, C = 49.7 (*p* = .19); anger-hostility: I = 48.8, C = 51.4 (*p* = .39); vigor: I = 50.1, C = 46, (*p* = .15); fatigue: I = 50.7, C = 53.3 (*p* = .35) and confusion: I = 48.8, C = 51.8 (*p* = .27). These baseline scores showed no significant difference however, after three months, a statistically significant difference was noted in vigor: I = 50.1, C = 49.8, (*p* = .02) and depression-dejection: I = 42.8, C = 50.4, (*p* = .02) with the intervention group experiencing an improvement in both scores.

A reduction in the EPDS was noted in three studies post-infant massage with no significant differences at baseline [54,56,57]. In O’Higgins et al’s. [57] study the mean EPDS score for the intervention group reduced from 13.19 to 9.29. The support group saw a lesser reduction from 13.81 to 11. Onozawa et al. [56] reported a considerable reduction in EPDS. The median EPDS score for the intervention group at baseline was 15 and for the control group 16, post-intervention the intervention group scored 5 (*p* = 0.03) in comparison to the control group who scored 10. Likewise, a superior reduction in EPDS scores for the intervention group compared to the control group [54]. At baseline, I = 11.52, C = 11.31 (*p* = 0.461) and post-intervention: I = 7.75, C = 9.20 (*p* = <0.001). In context, a review of EPDS validation studies warned against generalising cut off points of the EPDS scale, instead making EPDS cut offs culture specific [73,74]. A cut of level of 13 appeared to specifically identify women with stronger symptoms of PND while a cut off of 11 was more sensitive but less specific [73]. Furthermore Matthey [74] found that although a wide range of studies argue that cut off points should be culture specific, many studies disagree arguing this is not of importance. Oswalt and Biasini [58], further reported a significant reduction in depression post-infant massage according to the BDI II. At baseline: C = 7.67, I = 8.38 (*p* = .89), post-intervention: C = 8.61, I = 5.31 (*p* = .04). Women who participated in infant massage also experienced a reduction in parental distress from 21.25 at baseline to 20.75 post-intervention. However a reduction in parental distress was also observed in the control group (28.44 at baseline, 25 post-intervention), thus results of the intervention group may not be attributed to infant massage. Interestingly, women in the intervention group exhibited improved feelings relating to physical contact with their infant with post-intervention scores decreasing from 33.50 to 33.42. Contrastingly in the control group, feelings related to physical contact with their infant deteriorated over time from 26.56 to 31.85. Notably, control scores at baseline were lower than the intervention group and no improvement in maternal confidence was observed in either group.

Mindell et al. [59], reported improved sleep quality for women in the intervention group using infant massage with less night waking at the end of the study (Baseline: I = 2.47, C = 2.39, post-test: I = 1.86, C = 2.32), in addition to a reduction in the ESS (Baseline: I = 8.98, C = 9.49, post-test: I = 6.84, C = 8.71). Women in the intervention group experienced a significant improvement in mood (BMIS) (Baseline: I = 48.63, C = 50.98, Post-test: I = 52.39, C = 51.92) over the two week period. However, there was no significant reduction or difference in the PSI and STAI in both groups. Similarly, O’Higgins et al. [57] reported a small reduction in STAI scores for both intervention and control group however scores of both groups remain markedly higher than the non-depressed group.

### Women’s experiences of mother-led infant massage

Unequivocal outcomes relating to the experiences of women were evident in three studies [53,55,58]. All three studies reported increased maternal satisfaction levels during infant massage owing to positive cues received from infants i.e. smiling and eye contact. Additionally, structured class settings, calming atmospheres, engaging instructors and peer support contributed positively to maternal satisfaction levels [53,55]. Women appreciated the special time infant massage provided them:

“*I use infant massage to get some time for just the two of us*, *well actually*, *we spend all day together*, *but during the massage*, *I pay close attention to her"* [55, p.494].

*“I feel that the more frequent I do the massage for baby*, *the closer I am with baby”*[53, p.192].

Women also reported a reduction in feelings of guilt and an opportunity to experience calm in addition to increased self-confidence and self-esteem.

*“I felt like I was able to soothe her and that made me more confident in my role as a mother”* [55, p. 494].

*“My baby is more calm and relaxed*, *slept longer at night”* [53, p. 192].

Improvements in mother-infant interactions were also noted along with feelings of strengthened attachment. Compellingly:

*“I felt a loss of attachment to my baby*, *which I believe other mothers have*, *but somehow it felt like I resumed it again*, *in a way*, *while I was practicing infant massage”* [55 p. 494].

*“I get more attached to the baby because when I did the massage with baby*, *I talked to baby and have eye contact with him… felt the bonding is there”* [53 p. 192].

Women appreciated learning new skills in relation to caring for their baby and observing baby’s cues.

*“Sometimes baby refused*, *see his mood*, *I would stop*, *maybe he wanted milk*, *sleep or not comfortable*, *already know his cue*” [53 p. 192].

Women reported the importance of spousal support to attend baby massage and acquiring peer support at the group.

*“My older child is quite active*, *concerned he would disturb baby*, *my husband would help to look after the older child while I did baby massage*” [53 p. 192].

*“The best thing is*, *able to meet new people*, *other babies*, *exchange experience”* [53 p. 192].

Overall these interpretations suggest the potentially positive and meaningful effect infant massage can have on women and the positive influence it can have on maternal mood, confidence, self-efficacy and mother-infant interactions.

### The effects of mother-led infant massage on the mother-infant relationship

Although the effects of infant massage on symptoms of maternal postnatal depression is the primary aim of this review, it is important to acknowledge the additional benefits of infant massage that were identified. Women who attended infant massage classes reported more meaningful interactions with their infants [55].

“*It was a good way to communicate*, *she did not have a lot of facial expressions*, *I knew when she was content*”[55 p. 494].

“*I see that she loves it*, *she laughs and smiles a lot*” [55 p. 495].

As reported by Onosawa et al. [58], mother-infant interactions significantly improved for those practicing infant massage. Results include maternal response characterised by “warm to cold”; Baseline: Intervention (I) = 3.2, Control (C) = 3.2 and “non-intrusive to intrusive”; Baseline: I = 3.5, C = 4.0 vs post-intervention “warm to cold” I = 3.6, C = 3.2 and “non-intrusive to intrusive” I = 4.5, C = 3.2. Infant responses also demonstrated improvements in the overall interaction, with intervention group exhibiting substantially better results than the control group in the categories “attentive to non-attentive” Baseline: I = 1.3, C = 2.0 vs. final session: I = 3.0, C = 2.2; “lively to inert” Baseline: I = 2.0, C = 2.7 vs. final session: I = 4.0, C = 2.7, “happy to distressed” Baseline: I = 2.0, C = 3.0 vs. final session: I = 4.5. C = 3.5. Additionally, the longstanding benefits of infant massage was conveyed by O’Higgins et al. [57] where there was a statistically significant improvement in infant performance in interaction in the massage group at one year. Furthermore, Oswalt and Biasini [58], demonstrated a higher level of parent-child dysfunction for mothers with HIV in their control group compared to the massage group. Also, mothers with HIV in the intervention group displayed improved feelings about physical contact with their baby [58].

Lastly, infant massage has additional benefits for infants. Infants who experienced infant massage with their mother tended to have higher growth measurements [58], experienced fewer night arousals [59] and improved temperaments [57].

## Discussion

The aim of this systematic review was to examine the effectiveness of infant massage on symptoms of maternal postnatal depression. The findings were categorised under three themes; the effects of infant massage on symptoms of postnatal depression, women’s experiences of infant massage and additional benefits of infant massage. All eight included studies demonstrated a reduction in depressive symptoms in mothers using infant massage compared to those who received routine postnatal care, despite the various types of research and sample sizes used.

The difficulties experienced by women during their transition to motherhood is widely documented particularly in the context of expectation versus reality when becoming a mother [1,75]. Should these expectations not be realised, it may lead to feelings of inadequacy, guilt and failure, whilst a lack of awareness of psychological issues and insight into normal emotions after birth can shadow symptoms of PND [1]. Although all women are at risk of PND, many predictors do exist [76]. PHNs are ideally placed to identify women at risk of PND[450] and although PHNs are not frequently linked with women in the antenatal period, they visit women within 72 hours of discharge from hospital, thus can recognise women at risk of or experiencing symptoms of PND from an early stage. It is documented that women experiencing PND have challenges interacting with their infant [77]. Such challenges can result in adverse outcomes for both mother and baby. These include deteriorating mental and physical wellbeing for the mother [78] with an additional broad range of developmental issues for the infant if left undetected and untreated [77,79]. Infant massage classes provided over a number of weeks by a trained instructor can provide structured support for the mother-infant dyad, concentrating particularly on meaningful mother-infant interaction and provision of social support [53,55–57] This review demonstrated the positive effect infant massage may have on symptoms of postnatal depression [52–59], therefore the promotion of a PHN-led intervention such as infant massage may provide a blend of social and professional support which advocates for positive maternal wellbeing and positive parent-infant interactions.

Both formal and informal support can positively influence a woman’s mental health and self-efficacy when delivered appropriately [80]. In this review, women appreciated peer-support in infant massage classes when exchanging experiences, along with knowledgeable and informative instructors [53,55,58]. Women reported enhanced feelings of attachment, improved feelings of closeness to their baby and a sense of achievement in recognising their baby’s desires and dislikes. Low levels of maternal self-confidence and increased stress levels are common in new mothers, particularly first-time mothers [81]. PHN-led infant massage may help to relieve such symptoms using its two pronged approach of professional support and peer-support, aspiring to empower women [82].

In addition to reduced symptoms of PND, improved mother-infant interactions was observed in women using infant massage [55–56], with habitual infant interactions at one year [57]. Oswalt and Biasini [58], also reported a lower level of parent-child dysfunction in women who attended infant massage. This is significant because a correlation exists between maternal mental health and child health outcomes [83]. Delayed developmental milestones [84] and a woman’s inability to recognise their infant’s needs [85] can exist in women experiencing mental health issues. Child development theorists advocate that all basic needs of a child must be met [86] in addition to consistent love and affection in order to become emotionally secure [87]. Thus, it is essential that women and infants are cared for as a dyad so that appropriate services are engaged to ensure the best possible outcomes [88]. A PHN-led infant massage class may be a cost-effective solution to enhance mother-infant relations as women would have an opportunity to learn infant communication and could be supported to do so in a safe and welcoming environment. However, despite consistent research findings, there is insufficient evidence of infant massage interventions in healthcare policy [23–25].

The Irish Specialist Perinatal Mental Health Services Model of Care [25] was introduced in 2017 and acknowledged the role of primary care in the screening and the referral of women at risk of PND and recognised the ideal position of PHNs. Although further educational training for PHNs in perinatal mental health has been provided online, further developments have not occurred to implement PHN-led interventions. The results of this review suggests that PHN-led infant massage classes may be a cost effective means of providing both social and professional support to mothers and may in fact contribute to a reduction in symptoms of PND while promoting positive mother-infant interactions. Indeed some areas have implemented such initiatives already, however, they are not widespread. The National Health Service (NHS) Highlands “Infant Massage Instruction Good Practice Guidelines” [89], are the only clinical guidelines found incorporating infant massage. Nevertheless, the struggle in integrating mental health and community healthcare services globally is evident [90,91]. Reduced staffing levels, inadequate resource provision and negative attitudes of healthcare professionals are well known barriers which prohibit the implementation of interventions in primary care [92]. Furthermore time constraints, knowledge and skill deficits and professional confidence in dealing with perinatal mental health is echoed in two studies examining barriers to sufficient perinatal mental health care provision by PHNs [2,49]. The National Maternity Strategy 2016–2026 [24] called for better integration of mental healthcare with primary care while the Slaintecare Report [93] recommended adequately funded child health and wellbeing services in accordance with the National Healthy Childhood Programme [94] to support parents appropriately and ensure every child has the best start to life [94]. Alas, completed and integrated rollout is prolonged.

### Limitations

To the best of the review team’s knowledge, this is the first systematic review to examine the effects of infant massage on symptoms of maternal postnatal depression however the findings should be considered in line with the following limitations. Small sample sizes inhibited clear outcomes and a lack of qualitative studies limits the applicability of results. The inclusion of studies published only in the English language has potentially introduced a bias. Furthermore, as the primary researcher is an International Association of Infant Massage Instructor and a Public Health Nurse what was considered relevant to the research question may be influenced by perspective and training.

### Implications for practice

Clinical guidelines for the implementation of infant massage in primary care do not exist in Ireland and appear scarce worldwide. The NHS Highland “Infant Massage Instruction Good Practice Guidelines” [89] recognise the positive impact infant massage can have on the wellbeing of both women and infants. SIGN [22] advocate for additional interventions for women and infants experiencing poor attachment and interventions should be particularly aimed at the mother-infant relationship in an effort to manage symptoms of PND. The implementation of clinical practice guidelines in Ireland incorporating infant massage could potentially enhance outcomes for mothers.

### Recommendations for future research

This review acknowledges a paucity of research on the effect of infant massage on symptoms of maternal postnatal depression. Research on the effects of infant massage on symptoms of maternal postnatal depression is primarily quantitative. Further qualitative studies capturing women’s experiences of infant massage and its impact on their psychological wellbeing would be beneficial in order to conceptualise women’s experiences in relation to infant massage, implement further infant massage courses in primary care and to ensure adequate numbers of staff are trained to provide this service. In turn, further evidence may trigger the inclusion of interventions such as infant massage in primary care policies, particularly within the Public Health Nursing service.

## Conclusion

This systematic review examined the effectiveness of infant massage on symptoms of maternal postnatal depression. Previous research has concentrated on the effects of infant massage on bonding and attachment and the physiological advantages of infant massage on infants, rather than the direct impact of infant massage on women. Infant massage may reduce symptoms of depression, improve maternal sleep quality, reduce anxiety and stress levels, reduce feelings of guilt and improve maternal confidence, satisfaction and attitude towards physical contact with their infant. The promotion of an infant massage intervention in the Public Health Nursing service would advocate a more proactive approach to perinatal mental health and potentially improve outcomes for both women and their infants.

## Supporting information

S1 Checklist PRISMA 2020 checklist. (DOCX)
